# Supplementary material for: Genome Stability Is in the Eye of the Beholder: CR1 Retrotransposon Activity Varies Significantly across Avian Diversity
Source: Genome Biol Evol. 2021 Nov 22;13(12):evab259. doi: 10.1093/gbe/evab259 (PMC8665684; doi:10.1093/gbe/evab259)
Supplement: evab259_Supplementary_Data [file evab259_supplementary_data.zip › Galbraith_SI Information.docx]

**SI Information**

**Figures**

SI Figure 1. Phylogenetic tree of newly identified full length CR1s and full length avian CR1s from Repbase. The full length CR1s used are the centroids of order specific clusters constructed using VSEARCH at 90% identity. Phylogeny constructed using FastTree from a MAFFT alignment of the nucleotide sequences.

SI Figure 2. Scaled divergence of 3’ anchored CR1s identified in a) selected passerines and b) selected paleognaths. CR1s were initially identified using a reciprocal BLAST search based on libraries consisting of RepBase avian and crocodilian repeats and the centroids of full length sequences identified within the order clustered in VSEARCH.

SI Figure 3. Number of high confidence insertions of dominant CR1 families in owls approximated by presence/absence patterns of orthologous CR1 insertions between 100 and 600 bp in length. CR1 subfamilies are labeled by colour (see legend). Phylogeny adapted from Salter et al. [(2020)](https://paperpile.com/c/gCNh6p/vcINe/?noauthor=1).

SI Figure 4. Scaled divergence of 3’ anchored CR1s identified in species of Amazon parrot (*Amazona*). CR1s were initially identified using a reciprocal BLAST search based on a consisting of RepBase avian and crocodilian repeats and the centroids of full length sequences identified within parrots clustered in VSEARCH.

SI Figure 5. Presence/absence workflow. 3’ anchored CR1 insertions in a genome between 100 and 600 bp (1) were identified with BLASTN and had coordinates extended to include 600 bp of flanking sequence at both the 5’ and 3’ ends (2). The resulting 1300-1800 bp long sequences were searched for in a related genome using BLASTN. Hits containing the entire insertion and at least 150 bp of each flank were treated as ancestral insertions (3). Hits to insertion not containing any flanking region, with hits to the flanking sequence on differing strands or multiple hits to a single flanking sequence far from each other were treated as unresolvable and discarded. Insertions having at least 150 bp of each flank in close proximity and one flank containing at least 90 bp of the insertion were treated as ancestral insertions of which part was deleted in the species being searched (4). Sequences remaining were either flanks in close proximity or flanks plus a portion of the CR1 insertion. The distance between the flanks potentially containing part of the insertion was calculated in both species, qdist in the query species and sdist in the related species (5). If qdist was greater or equal to the length of the original CR1 insertion (olen) minus the length of 3x the 3’ microsatellite monomer and sdist was within the length of 2x the 3’ microsatellite monomer the insertion was treated as since divergence (6). If qdist was within the length of 2x the 3’ microsatellite monomer and the sdist was greater than 90 bp the insertion was treated as ancestral (7). Any insertions not fitting these criteria were treated as unresolvable and discarded. This strict process was calibrated through adjusting variables and viewing resulting pairwise alignments between regions identified as orthologous, using the presence of target site duplications in the query species and if part of the CR1 insertion was present in the related species to determine if insertions had truly occurred in an orthologous region, erring on the side of discarding new insertions over misclassifying partially deleted ancestral insertions as new insertions.

SI Figure 6. Presence/absence resolution - Example of the method we used to resolve the presence/absence, and hence insertion timing, of each CR1 in a species (species a), two related species (species b and c) and an outgroup (species d). The CR1 insertion in question is represented in green, the flanking regions in black and the branches labelled 1-3. The branch in bold italics is the branch on which the insertion occurred. If an CR1 was present in species a through c we considered the repeat to have been inserted at branch 1 (i), if in a and b at branch 2 (ii) and if in species a alone to be since the divergence from the immediate sister species and on branch 3 (iii). If present in all three species and the outgroup species examined we consider the repeat to be ancestral (iv). If a CR1 was absent from an immediate sister species but present in the more distant related species we considered this to be a result of deletion in the immediate sister species (v). Finally, if the orthologous region was present in a species or group of species but could not be resolved in the immediate sister species we considered the timing of insertion to be unresolvable (vi).

**Tables**
SI Table 1. Genome assemblies used throughout this analysis. All genomes were downloaded from GenBank.

SI Table 2 - Reclassification of previously described full length avian CR1s based on their position within our CR1 phylogeny (SI Figure 1; same color coding).

SI Table 3. Resolution of presence or absence of orthologous CR1 insertions between 100 and 600 bp in related species in waterfowl, shorebirds, perching birds, parrots, owls, and kiwis + cassowary + emu genomes. Cells highlighted in yellow are the values used to construct Figures 4 and 5 and SI Figure 3.

**Data**

SI Data 1 - Coordinates of full length CR1s identified in each genome in BED format. For the appropriate genome version see SI Table 1.

SI Data 2 - Multiple sequence alignment used to create the CR1 phylogeny (SI Figure 1) and Newick tree of said phylogeny.

SI Data 3. Divergence plots of coverage of 3’ anchored CR1s identified in each species of bird. CR1s were identified using a reciprocal BLAST search based on libraries consisting of Repbase avian and crocodilian repeats and the centroids of full length sequences identified within the order clustered in VSEARCH. Jukes-Cantor distance was calculated from the reciprocal BLAST search output.

SI Data 4. Divergence plots of number of 3’ anchored CR1s identified in each species of bird. CR1s were identified using a reciprocal BLAST search based on libraries consisting of Repbase avian and crocodilian repeats and the centroids of full length sequences identified within the order clustered in VSEARCH. Jukes-Cantor distance was calculated from the reciprocal BLAST search output.

SI Data 5. Transposition in transposition plots of CR1s identified in the waterfowl, shorebird, parrot, kiwi, cassowary and emu genomes. CR1s were masked with RepeatMasker [(Smit 2004)](https://paperpile.com/c/gCNh6p/odX2) and plots constructed with TinT [(Churakov et al. 2010)](https://paperpile.com/c/gCNh6p/iquI).
