# Supplementary figures and images for: Genome Stability Is in the Eye of the Beholder: CR1 Retrotransposon Activity Varies Significantly across Avian Diversity
Source: Genome Biol Evol. 2021 Nov 22;13(12):evab259. doi: 10.1093/gbe/evab259 (PMC8665684; doi:10.1093/gbe/evab259)

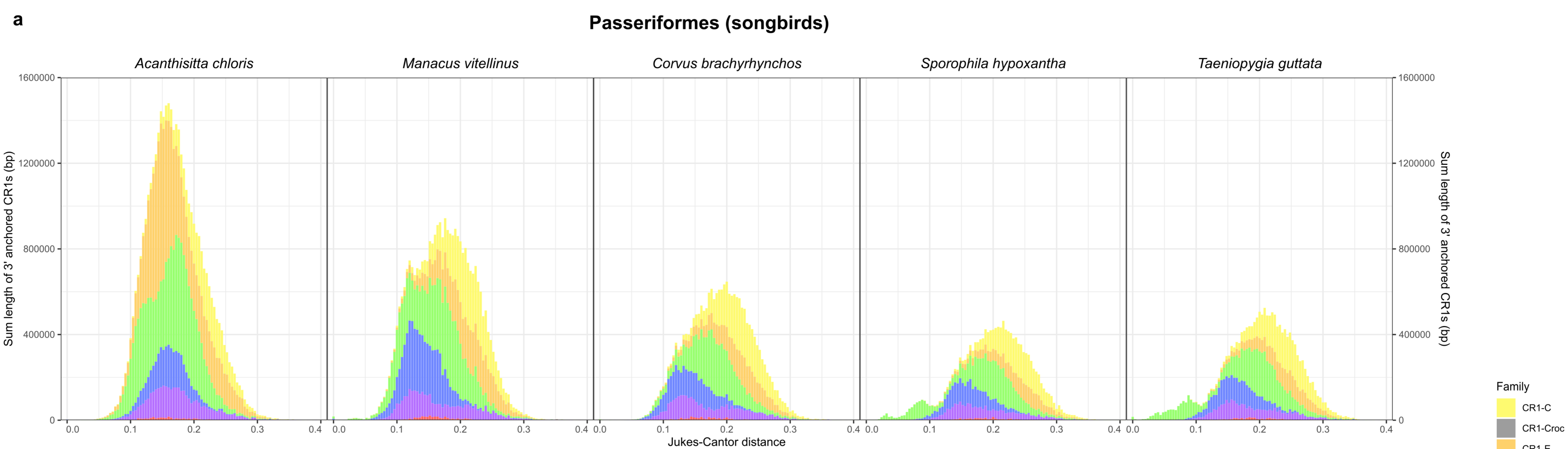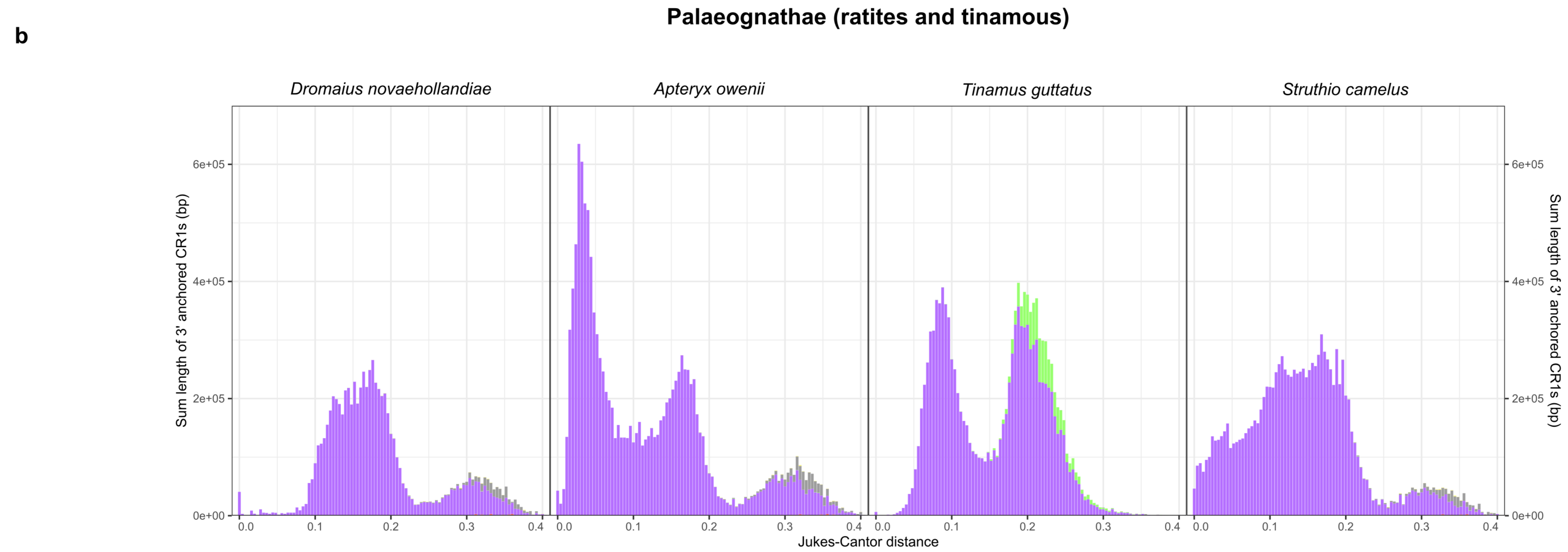

● CR1-C  
 ● CR1-E  
 ● CR1-J  
 ● CR1-X  
 ● CR1-Y  
 ● CR1-Z

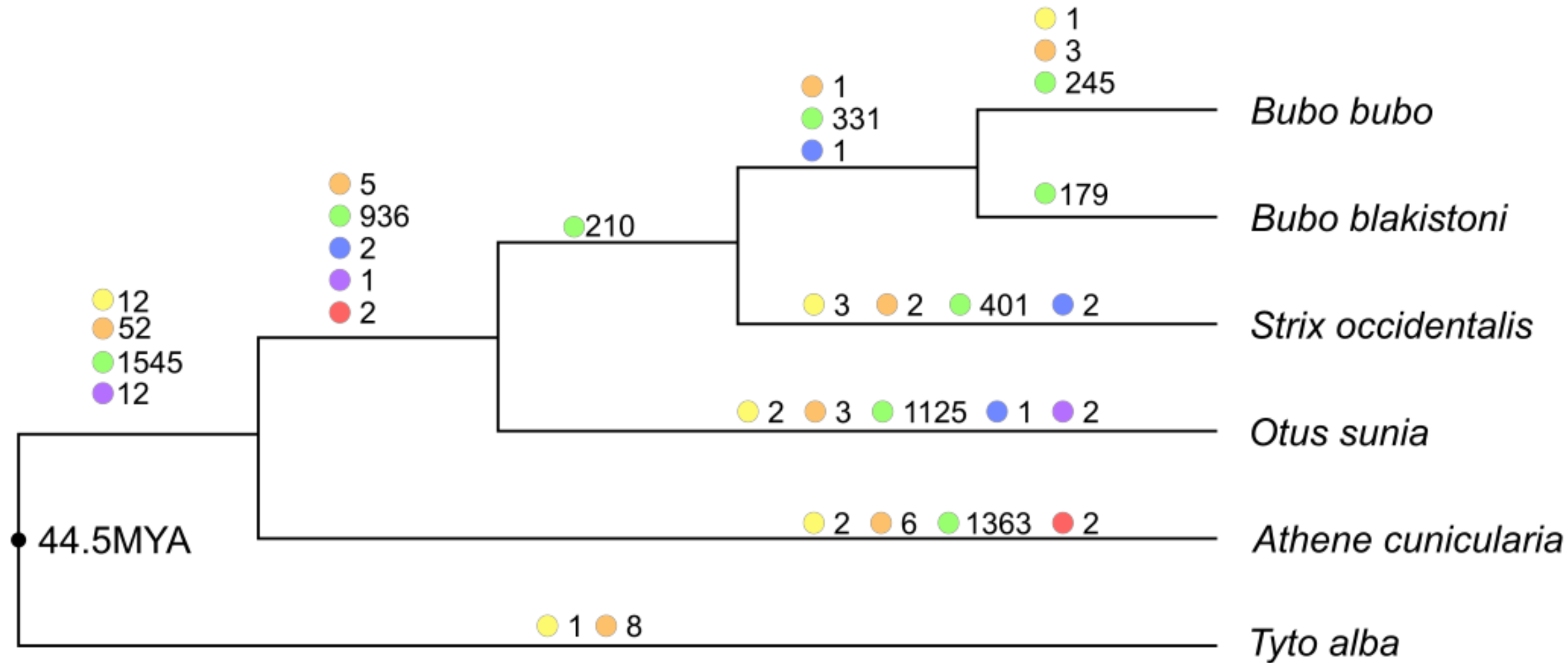

*Amazona vittata*

*Amazona collaria*

*Amazona aestiva*

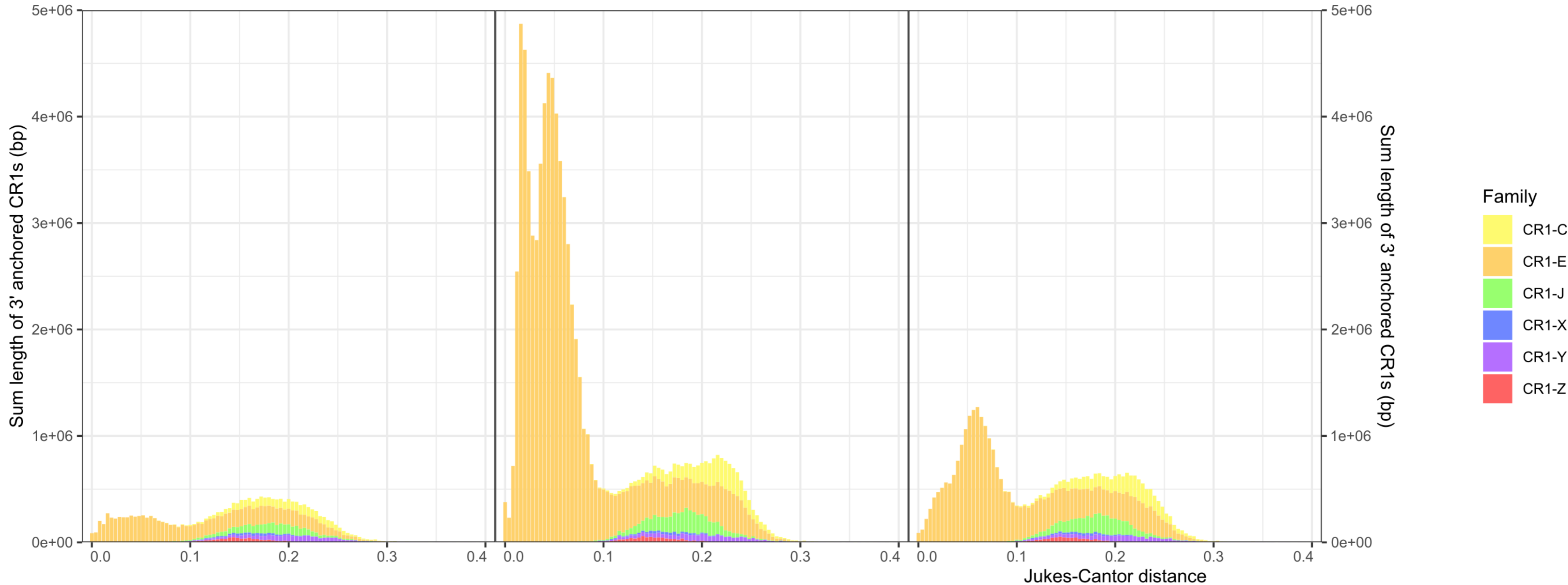

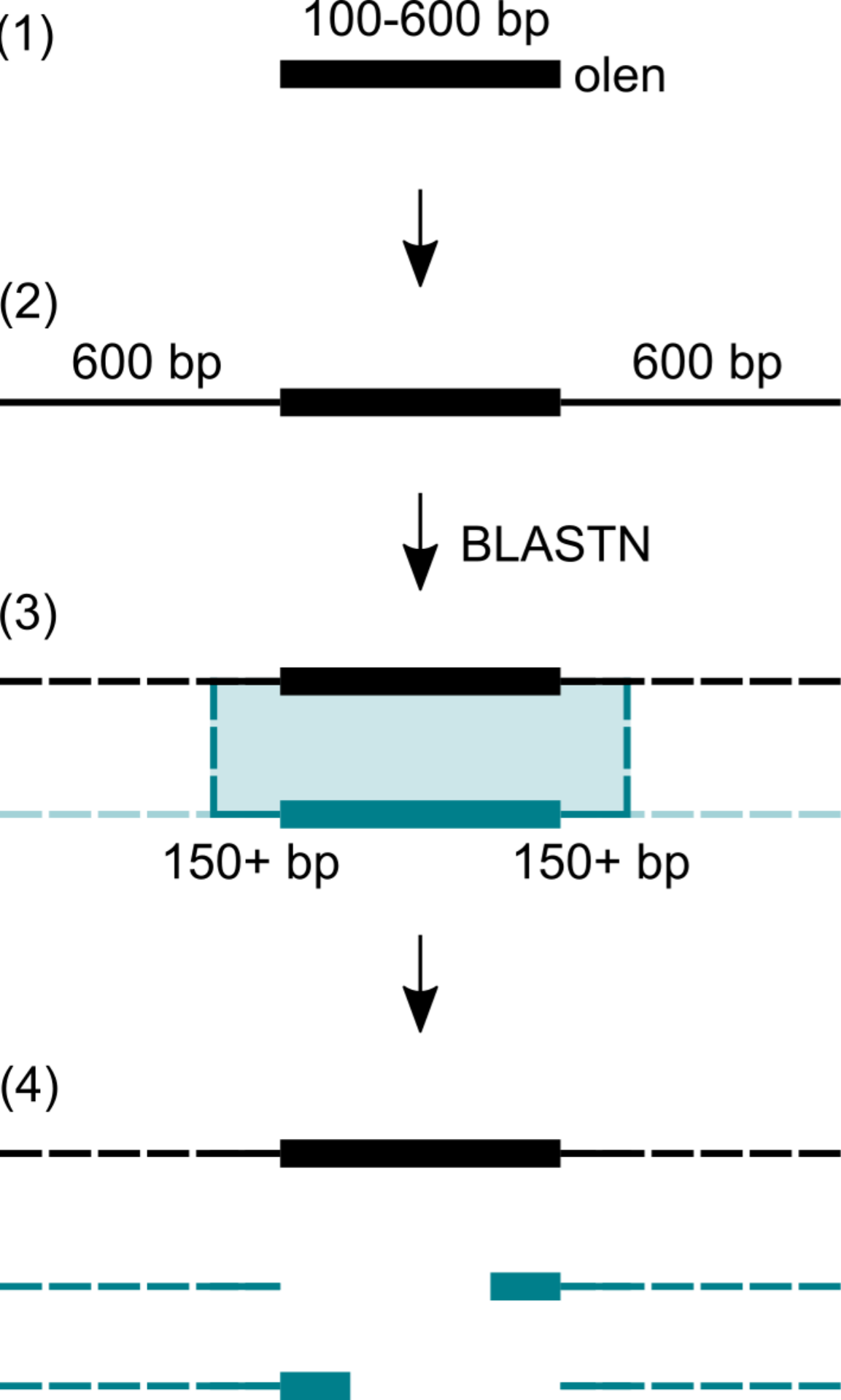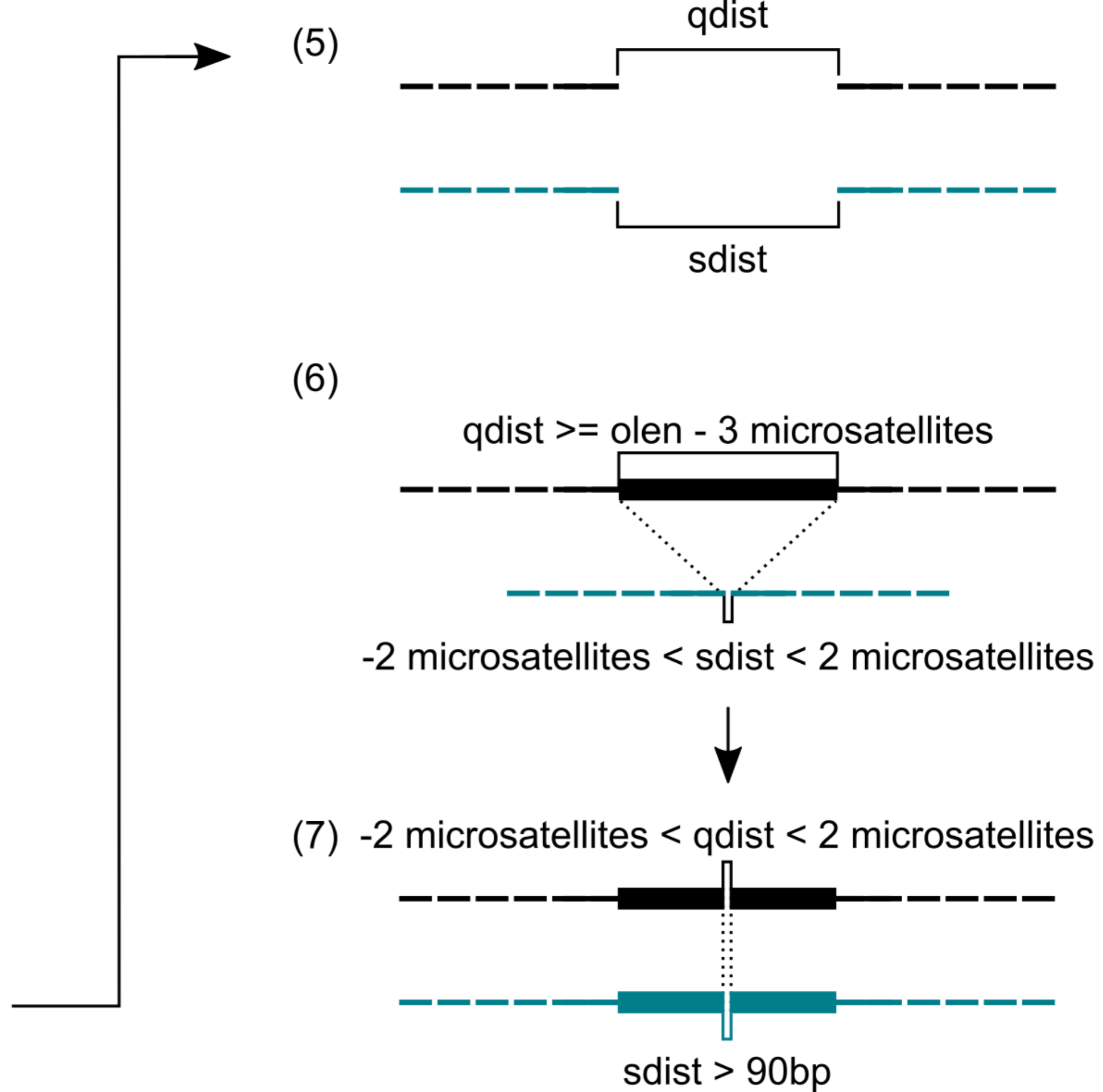

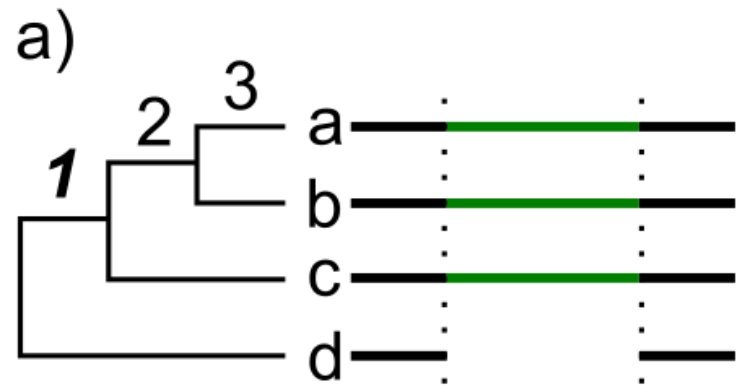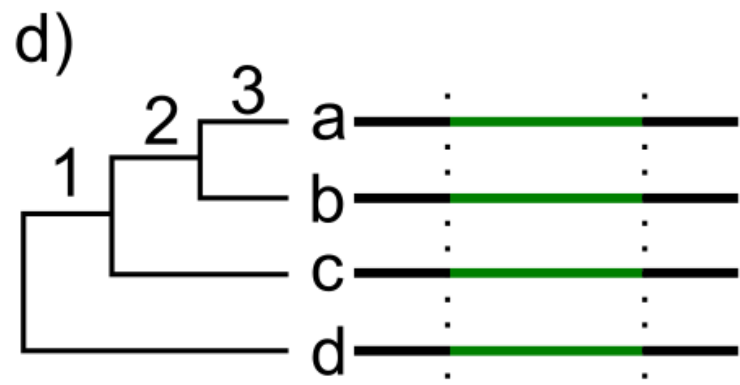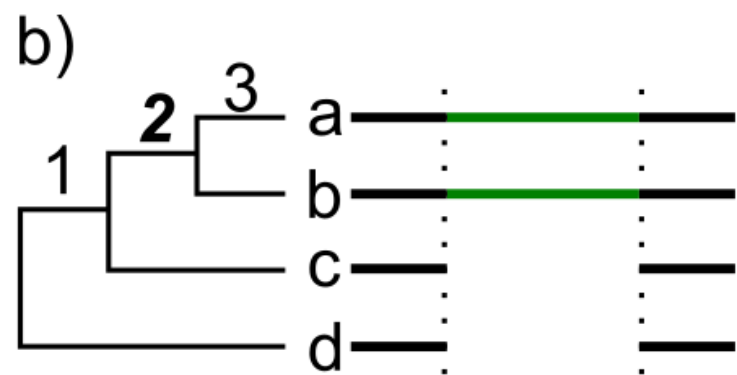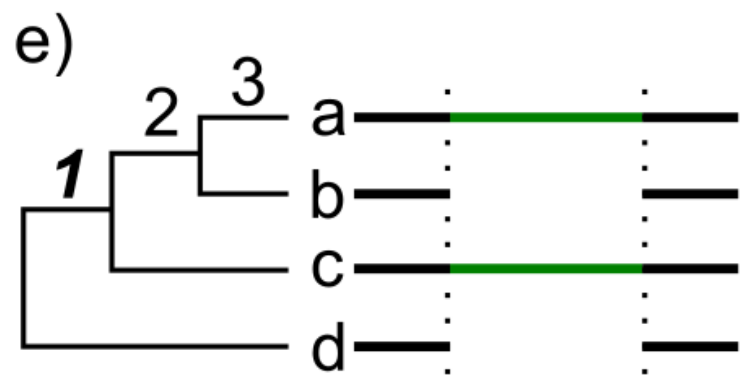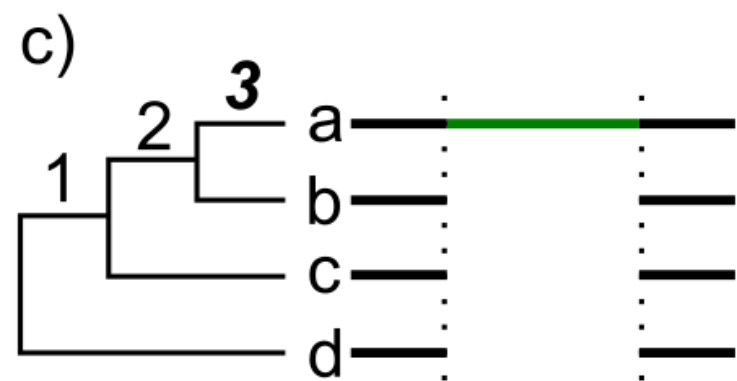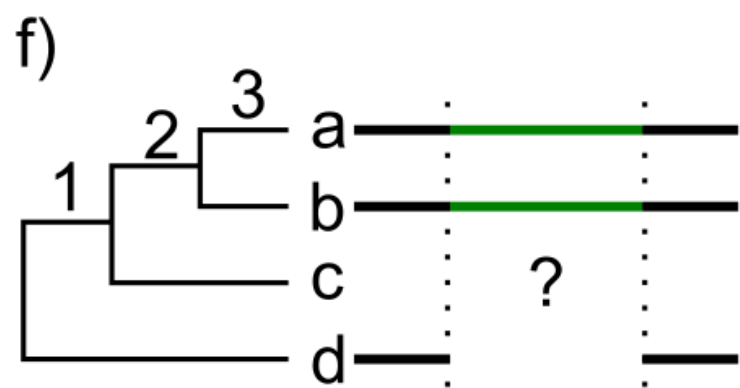

Supplement: evab259_Supplementary_Data [file evab259_supplementary_data.zip › Galbraith_GBE_revision_SI_Figs_2-6.pdf]

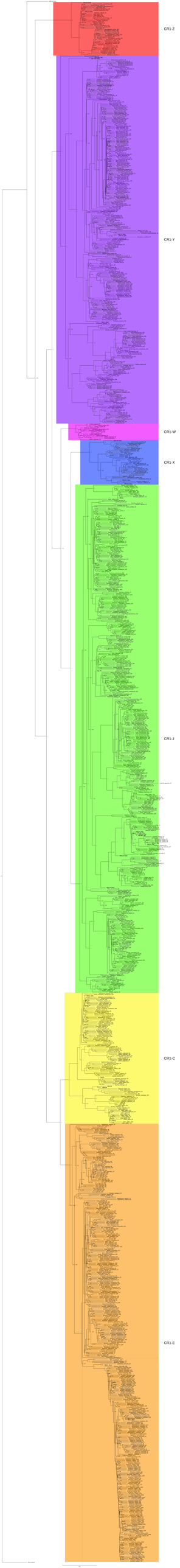

Supplement: evab259_Supplementary_Data [file evab259_supplementary_data.zip › Galbraith_GBE_revision_SI_Fig_1_RT_EN_90_expanded.tif]
